# Endoscopic Submucosal Dissection (ESD) for the Management of Fibrotic Non-Lifting Colorectal Lesions (NLCLs): Results from a Large Multicenter Retrospective Study

**DOI:** 10.3390/cancers17071242

**Published:** 2025-04-06

**Authors:** Giuseppe Dell’Anna, Ernesto Fasulo, Paolo Cecinato, Giovanni Barbara, Alberto Barchi, Edi Viale, Dario Esposito, Simone Grillo, Romano Sassatelli, Alberto Malesci, Sara Massironi, Vito Annese, Lorenzo Fuccio, Antonio Facciorusso, Gianfranco Donatelli, Silvio Danese, Francesco Azzolini

**Affiliations:** 1 Gastroenterology and Gastrointestinal Endoscopy, IRCCS San Raffaele Hospital, 20132 Milan, Italy; fasulo.ernesto@hsr.it (E.F.); barchi.alberto@hsr.it (A.B.); viale.edi@hsr.it (E.V.); esposito.dario@hsr.it (D.E.); malesci.alberto@hsr.it (A.M.); danese.silvio@hsr.it (S.D.); azzolini.francesco@hsr.it (F.A.); 2 Gastroenterology and Gastrointestinal Endoscopy, IRCCS Policlinico San Donato, 20097 San Donato Milanese, Italy; vito.annese@grupposandonato.it; 3 Faculty of Medicine and Surgery, Vita-Salute San Raffaele University, 20132 Milan, Italy; massironi.sara@hsr.it; 4 Gastroenterology and Digestive Endoscopy Unit, Arcispedale Santa Maria Nuova di Reggio Emilia, AUSL-IRCCS di Reggio Emilia, 42123 Reggio Emilia, Italy; paolo.cecinato@gmail.com (P.C.); simone.grillo@ausl.re.it (S.G.); romano.sassatelli@ausl.re.it (R.S.); 5 Gastroenterology Unit, IRCCS Azienda Ospedaliero-Universitaria di Bologna, 40138 Bologna, Italy; 6 Gastroenterology Unit, Istituti Ospedalieri Bergamaschi, 24046 Bergamo, Italy; giovanni.barbara@unibo.it; 7 Department of Medical and Surgical Sciences (DIMEC), University of Bologna, 40100 Bologna, Italy; lorenzofuccio@gmail.com; 8 Faculty of Medicine and Surgery, University of Salento, 73100 Lecce, Italy; antonio.facciorusso@virgilio.it; 9 Clinical Effectiveness Research Group, University of Oslo, 0316 Oslo, Norway; 10 Unité d’Endoscopie Interventionnelle, Hôpital Privé des Peupliers, 75013 Paris, France; donatelligianfranco@gmail.com; 11 Department of Clinical Medicine and Surgery, University of Naples Federico II, 80138 Naples, Italy

**Keywords:** EMR, ESD, NLCL, hybrid, colorectal, polyps, recurrent, no-lifting

## Abstract

**Background/Objectives**: The management of non-lifting colorectal lesions (NLCLs), often resulting from previous unsuccessful treatments or biopsies, remains challenging due to submucosal fibrosis that prevents adequate lifting. Endoscopic submucosal dissection (ESD) is a viable option for achieving complete resection in such cases. However, when standard ESD is not feasible, conversion to hybrid ESD (H-ESD) has been proposed as a rescue strategy. This study aimed to assess ESD’s feasibility, effectiveness, and safety for NLCLs, including cases requiring conversion to H-ESD, when performed by experienced endoscopists in tertiary referral centers. **Methods**: In this multicenter retrospective study, data from patients with NLCLs treated by ESD/H-ESD between January 2009 and September 2022 were analyzed. The primary endpoint was the recurrence rate (RR). Secondary endpoints included the adverse event (AE) rate, technical success (TS) rate (en bloc resection regardless of technique), complete resection (CR), curative resection (cR) rates, and surgical intervention rate. Predictors of ESD technical success were identified. **Results**: In total, 178 patients with NLCLs were included (52 previously biopsied, 126 recurrent after previous resection). ESD was used in 111 (62.4%) and H-ESD in 67 (37.6%) cases. During a median follow-up of 373 days (IQR 540), the overall RR was 3.6%. The overall AE rate was 13.4%, and perforation was the most frequent (8.4%). All AEs were successfully managed endoscopically. The TS rate was 71.9%, significantly higher in previously biopsied lesions compared to recurrent ones (78.8% vs. 55.6%, *p* = 0.04). On multivariate analysis, rectal location (*p* < 0.001), F1 fibrosis (*p* = 0.026), and previously biopsied lesions (*p* = 0.006) predicted ESD TS without the need for conversion to H-ESD. **Conclusions**: ESD/H-ESD is feasible and safe for NLCLs when performed by experienced operators, with low RR amenable to endoscopic treatment. Previously biopsied lesions, rectal location, and low fibrosis predict ESD TS.

## Simple Summary

The treatment of colorectal lesions, which do not lift easily due to fibrosis caused by previous treatments or biopsies, is a significant challenge for endoscopists. Endoscopic submucosal dissection (ESD) is an advanced method that allows for the complete removal of these lesions in a single procedure, but it can be technically difficult and time-consuming. To address this, a hybrid version of ESD (H-ESD) combines two techniques to make the procedure safer and faster as a backup option. In this study, we analyzed the effectiveness and safety of ESD and H-ESD across two centers to understand their outcomes and identify which factors predict success. Our findings provide valuable insights to improve treatment strategies and reduce the need for surgery in patients with such complex colorectal lesions.

## 1. Introduction

**Figure 1:**
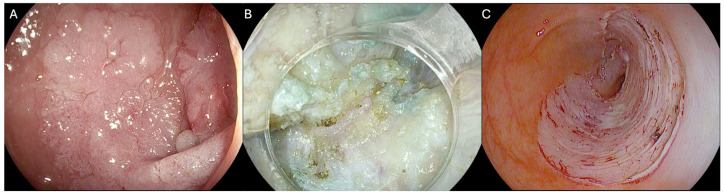
Endoscopic images showing (**A**) recurrent non-lifting colorectal lesion (NLCL) of the sigmoid colon; (**B**) fibrosis during endoscopic submucosal dissection; and the (**C**) post-resection site. The copyright of the image belongs to the authors.

The management of non-lifting colorectal lesions (NLCLs) represents a significant challenge in endoscopy [1]. Such lesions, often resulting from previous unsuccessful treatment attempts or biopsies, are difficult to treat due to submucosal fibrosis that prevents adequate lifting [2]. The presence and degree of submucosal fibrosis can significantly impact the feasibility, procedural time, clinical outcomes, and safety profile of the endoscopic treatment [3,4,5]. Various endoscopic techniques have been proposed for the management of NLCLs; however, to date, no standard approach has been established. Endoscopic submucosal dissection (ESD) is a viable option in such cases, owing to its ability to achieve en bloc and curative outcomes in a single procedure [6,7] (Figure 1). Nevertheless, ESD is an advanced technique that necessitate substantial training and expertise [8,9,10]. Moreover, it is associated with a considerable risk of complications and may require longer procedure times [11].

**Figure 2:**
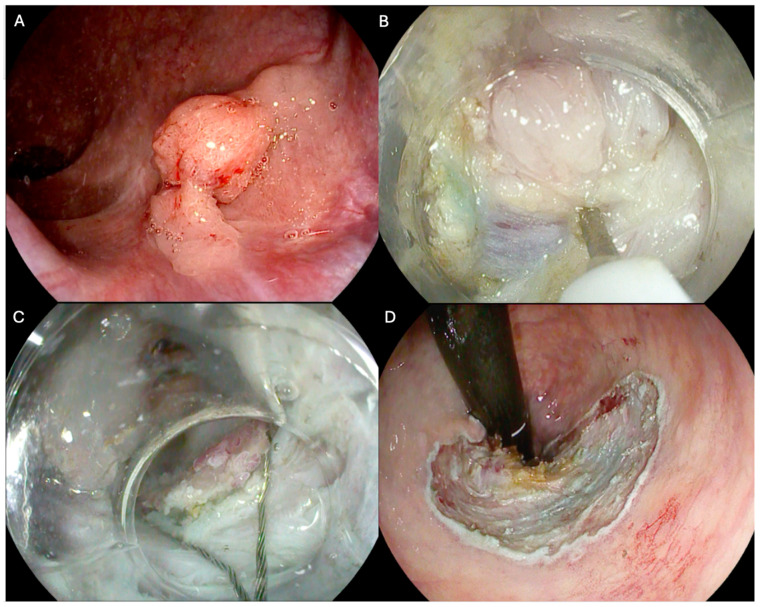
Endoscopic images showing (**A**) recurrent non-lifting colorectal lesion (NLCL) of the distal rectum; (**B**) fibrosis during endoscopic submucosal dissection; (**C**) conversion to hybrid endoscopic submucosal dissection; and the (**D**) post-resection site. The copyright of the image belongs to the authors.

To address the challenges encountered in the management of NLCLs, hybrid-ESD (H-ESD) has been proposed as a rescue therapy [12,13,14,15]. H-ESD combines the principles of conventional ESD and endoscopic mucosal resection (EMR). The procedure involves submucosal injection, followed by knife incision and partial dissection to create a mucosal flap, which is then resected using a snare [16,17]. This approach, also used as rescue therapy, aims to achieve en bloc resection while minimizing fragmentation and reducing the overall procedural time required for complete excision in a single session (Figure 2).

Given these considerations, we conducted a multicenter retrospective study to assess the feasibility, effectiveness, and safety of ESD and H-ESD for the treatment of NLCLs when performed by experienced endoscopists in tertiary referral centers. We aimed to evaluate the role of these techniques in the management of NLCs and to provide insights into their clinical outcomes and associated risks in a real-world setting.

## 2. Materials and Methods

### 2.1. Study Population

We performed a retrospective analysis of prospectively collected data of all consecutive patients diagnosed with NLCLs with submucosal fibrosis at IRCCS San Raffaele Hospital in Milan (Italy) and Arcispedale Santa Maria Nuova in Reggio Emilia (Italy) from January 2009 to September 2022. Data were collected into a prospective registry, approved by the local Ethical Committee (Protocol ID: REG_EGDS COLON; Approval Code: 79/12/2022). This observational study was designed and reported following the STROBE (STrengthening the Reporting of OBservational studies in Epidemiology) statement guidelines for reporting observational studies [18]. Inclusion criteria were colorectal lesions presenting the no-lifting sign due to previous endoscopic resection attempts or to previous biopsy sampling, age > 18 years, and lesions endoscopically treatable with ESD according to the most recent guidelines in that period [19,20]. Exclusion criteria were age < 18 years, lesions showing endoscopic features suspected of deep submucosal/muscular invasion or histological report of neoplastic lesion with surgical indication, lesions previously treated with ESD or H-ESD, and lesions unsuitable for ESD indication. The fibrosis degree of lesions was classified during the index procedure according to a semiquantitative classification: F1 or mild, characterized by a white web-like structure in the blue submucosal layer, and F2 or severe, as a white muscular-like structure without a blue transparent layer in the submucosal layer [21].

### 2.2. Study Endpoint

The primary endpoint of this study was the recurrence rate (RR). Recurrence was defined as any endoscopically visible and histologically confirmed adenomatous tissue at the previous resection site detected during any surveillance colonoscopy (SC). Confirmation was obtained either through biopsies or an examination of the removed specimen if immediate treatment was performed. Secondary endpoints included the technical success rate (TS), defined as en bloc resection with either ESD or H-ESD. Technical failure was defined as the need for piecemeal resection. Additionally, we evaluated the technical success rate specifically for standard ESD, defined as en bloc resection without conversion to H-ESD. This study also assessed the rate of adverse events (AEs), stratified by fibrosis cause and technique used, according to the AGREE classification [22]. Complete resection (CR) was defined as an R0 en bloc resection with histologically tumor-free lateral and deep margins [20]. Curative resection (cR) was defined as an R0 resection with submucosal invasion < 1000 microns from the muscularis mucosae, no lymphatic invasion, no vascular involvement, no budding, and poorly differentiated components [20]. For cases where histopathological reports lacked this information, slides were re-evaluated by an expert gastrointestinal pathologist at each of the two centers. Finally, the rate of surgical intervention at any time during the follow-up period was also included.

### 2.3. Endoscopic Procedure

All ESD procedures were conducted under deep sedation with anesthesiologic assistance or general anesthesia, according to local protocol. Different endoscopic systems were used according to the availability of the centers: Olympus (Olympus, Tokyo, Japan); Fujifilm (Fujifilm Tokyo, Japan); Pentax (Hoya Corp., Tokyo, Japan), using standard colonoscopes or gastroscopes for rectal lesions. The scopes were equipped with a distal transparent hood. CO_2_ insufflation was used in every procedure. All ESD procedures followed the basic principles indicated in ESGE guidelines [19,20]. The dissection strategy (standard, pocket, tunneling) and whether to apply traction systems were left to the discretion of the endoscopist. H-ESD completion involved using different-sized snares as needed. All procedures were performed by expert operators, defined as those having completed at least 100 colorectal ESDs and who regularly receive referrals from other hospitals, performing at least 25 ESDs per year to maintain proficiency throughout the entire study period [23].

### 2.4. Data Collection

Preprocedural clinical and demographic data included age, sex, and anesthesiologic risk classification according to the American Society of Anaesthesiologists (ASA) Physical Status Classification [24]. The lesions were analyzed based on the cause of fibrosis (recurrence vs. previously biopsied) and the treatment modality (ESD or H-ESD). Intraprocedural data included lesion localization, lesion long-axis and short-axis length (mm), area (cm^2^) calculated using the ellipse formula (short-axis length × long-axis length × 0.25 × 3.14), lesion morphology classified according to Paris’ endoscopic classification [25], glandular pattern according to Kudo’s classification [26], fibrosis degree, reason for fibrotic development, procedure time (min), and procedure speed (mm^2^/min), and the type of endoscopic devices used was also recorded. Post-procedure data included histology of the resected specimen, en bloc or piecemeal resection, radicality of the resection, need for surgery, recurrence, and mean follow-up in days and all follow-up SCs. Conversion to H-ESD was performed when, at the operator’s discretion, standard ESD proved unfeasible due to severe fibrosis, unstable position, or difficult access [27]; uncomfortable knife inclination; hemodynamic instability; or perforation. The reason for conversion was registered. Data on AEs were also collected. For any intraprocedural bleeding that required a dedicated endoscopic treatment, considerably lengthening the procedural time was considered; self-limiting or promptly treated bleeds were not recorded as AEs. Delayed bleeding was defined as overt hematochezia or melena arising from the resection site during the 6 *h* after completion of colorectal ESD. Full-thickness damage of the muscular layer with visualization of intra-abdominal space, mesenteric or mesorectal fat, was considered an intraprocedural perforation. Delayed perforation was defined as the appearance of radiological signs of perforation after colorectal ESD achievement, in an otherwise uneventful procedure. Two reporting systems (Endoxweb, Tesigroup, Italy, and OlyEndo, Unicode, Italy) were implemented in both centers involved in the study since 2019, providing electronic case report forms (CRFs) for patients and requinig mandatory inputs to complete the procedure report.

### 2.5. Statistical Analysis

Statistical analyses were performed using SPSS 26.0 (SPSS Inc., Chicago, IL, USA). Normality was assessed for the main demographic and descriptive variables using the Kolmogorov–Smirnov normality test, with the generation and evaluation of Q-Q plots assuming a 1-sided *p* value > 0.05 as an assumption of the normal distribution of the variable, implying the rejection of the null hypothesis. Concomitantly, heteroskedasticity was assessed with the Levene test. Continuous variables were later expressed as mean ± standard deviation (SD) or median and Interquartile Range (IQR) according to the normality test results. Median and range measures were provided for descriptive purposes. Categorical variables were expressed descriptively as numbers and percentages. Categorical parameters were compared using Pearson’s Chi-square test and Fisher’s exact test, along with Bonferroni correction for more than 2 groups within a variable, while continuous variables were compared using Pearson’s or Spearman’s correlation according to the normality distribution, with a Student's *t*-test or ANOVA test between 2 or more groups for normally distributed data, and with a Mann–Whitney test or Kruskar Wallis test between 2 groups for non-normally distributed data. The main outcomes, the CR, cR, technical success of the ESD procedure (or the “en bloc” resection rate or H-ESD conversion rate), surgery occurrence, and AE rate, were thoroughly calculated, and univariate analysis was performed to spot correlation with the most relevant independent variables. A logistic regression model was used to assess the impact of time on the probability of conversion to H-ESD. Model fit was assessed using the Hosmer and Lemeshow test, and the discriminative ability was evaluated through the area under the ROC curve (AUC). To identify a prediction model for primary and secondary endpoints, multivariate logistic regression models were built with a backward stepwise procedure, checking for multicollinearity (tolerance factor and variance-inflating factor calculation) for continuous independent variables. A *p* value < 0.05 in the univariate analysis was examined in the multivariate logistic regression models. Odds ratios (ORs) and 95% confidence intervals (CIs) were calculated using a logistic regression analysis. Two-sided *p* values < 0.05 were considered statistically significant.

## 3. Results

### 3.1. Baseline Characteristics

**Figure 3:**
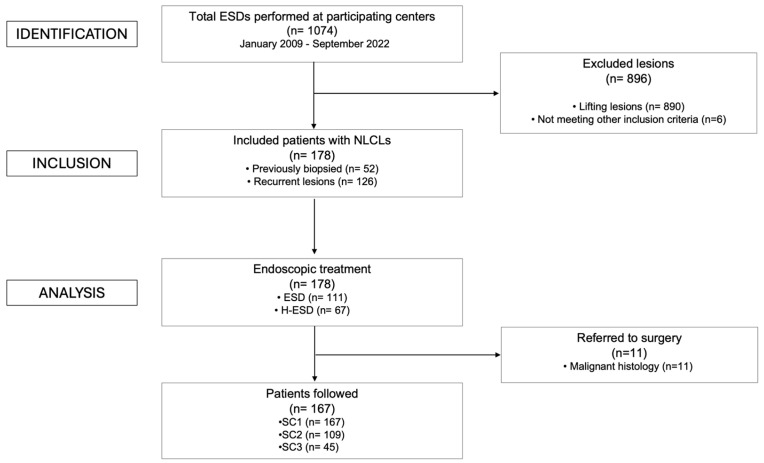
Study flow diagram according to the STROBE statement. ESD = endoscopic submucosal dissection; NLCL = non-lifting colorectal lesion; H-ESD = hybrid endoscopic submucosal dissection; SC = surveillance colonoscopy.

A total of 178 patients with 178 fibrotic NLCLs were included in this study (Figure 3). Fibrosis was due to previous biopsies in 29.2% of cases and to recurrences after previous endoscopic resections in 70.8% of cases. Among the patients included, 111/178 (62.4%) underwent ESD, while 67/178 (37.6%) required conversion to H-ESD. Conversion to H-ESD was mainly performed for severe fibrosis (62.7%) or difficult endoscopic access (i.e., very unstable position and uncomfortable knife inclination that could not be improved through endoscopic maneuvers or by changing patient’s decubitus position) (28.3%) (Supplementary Table S3). The median procedure time was 80 min (IQR, 60) for the overall cohort. The dissection speed was similar between the groups, with a median of 6.1 mm^2^/min (IQR, 8.1). Low-grade dysplasia (LGD) and high-grade dysplasia (HGD) were observed in 86.0% of cases, adenocarcinoma invading the submucosa (sm2-sm3) was found in 5.6% of cases, while other histologic findings were present in 2.3% of cases. Baseline characteristics are summarized in Table 1 and Supplementary Table S1.

### 3.2. Primary Endpoint

During a median follow-up of 373 days (IQR 540), the overall RR was 3.3%, with a total of six recurrences observed at the first surveillance colonoscopy (SC1) (median, 190 days; IQR, 240) among 167 patients, after excluding those referred for surgery (Table 2). Recurrent lesions showed a significantly higher rate of F2 fibrosis (68.3% vs. 30.8%, *p* < 0.01) and larger dimensions compared to previously biopsied, both in terms of the long axis (35 vs. 30 mm, *p* = 0.006), short axis (25 vs. 20 mm, *p* = 0.035), and area (7.1 vs. 4.7 cm^2^, *p* = 0.011). Recurrence was predominantly found in previously biopsied lesions (5/6 cases) and following H-ESD procedures (4/6 cases). Treatment at SC1 included EMR (2/6 cases), hot-biopsy avulsion (HBA) [28] (2/6 cases), ESD (1/6 cases), and H-ESD (1/6 cases), with a predominance of LGD lesions (83.3%). Importantly, two lesions treated for recurrence at SC1 were found to have recurred at SC2 (median, 390 days; IQR, 405). One of these, initially treated with EMR, was re-treated with ESD, while the other one, initially managed with H-ESD, was re-treated by EMR, due to its small size. At SC3 (median, 957 days; IQR, 953), no residual tissue was found. Late recurrences were equally distributed between biopsied and recurrent lesions and between ESD and H-ESD procedures, all showing LGD. Notably, all recurrences were successfully managed endoscopically without the need for surgical intervention.

### 3.3. Secondary Endpoints

The TS rate was 71.9% (128/178) for the overall cohort (Table 3). When considering only the traditional ESD approach, the en bloc resection rate was 62.4% (111/178). The TS was significantly higher in previously biopsied lesions compared to recurrent ones (82.7% vs. 67.5%, *p* = 0.04); conversely, the H-ESD conversion rate was higher in recurrent lesions (44.4% vs. 21.2%, *p* = 0.004) (Table 3). Severe fibrosis was the most frequent cause of technical failure (62.7%), followed by difficult endoscopic access (28.3%) (Supplementary Table S2). The CR and cR rates were similar between the previously biopsied and previously treated groups (90.7% vs. 93.0%, *p* = 0.475 for both). When CR was not achieved, the primary factor was deep margin involvement with sm2 histology, observed in 8/9 cases (88.9%). A single specimen exhibited lateral margin involvement, with histological analysis revealing sm1 invasion; this occurred post ESD treatment. The patient was subsequently referred for surgery due to the concomitant presence of lympho-vascular invasion.

### 3.4. Safety

Overall, the AE rate was 13.5% (24/178), with perforation being the most common complication, occurring in 8.4% (15/178) of cases. Bleeding was observed in 2.8% (5/178) of procedures, and one case reported both perforation and bleeding. All AEs were successfully managed endoscopically, with no cases requiring surgical intervention, as perforations were managed using clips or a clip-looping technique, while bleeding was controlled through mechanical hemostasis or thermal using bipolar forceps (Table 4). Moreover, according to the AGREE classification [22] (Supplementary Table S4), 3.3% (6/178) of patients experienced Grade I AEs, 8.9% (16/178) Grade II, and 1.7% (3/178) Grade III AEs (all Grade IIIa). No Grade IV or V complications were reported (Supplementary Table S5). Importantly, no statistically significant differences were observed in AE rates between the ESD and H-ESD groups (15.3% vs. 10.4%, *p* = 0.532), or between the groups with prior biopsy and recurrence (11.5% vs. 7.1%, *p* = 0.197).

### 3.5. Univariate and Multivariate Analysis

Univariate analysis identified lesion localization in the rectum (OR, 0.10; 95% CI, 0.03–0.28; *p* < 0.001), the presence of F1 fibrosis (OR, 0.12; 95% CI, 0.03–0.44; *p* = 0.0017), previously biopsied lesions (OR, 0.33; 95% CI, 0.58–0.86; *p* = 0.04), and the dimension of the lesion long axis (OR, 0.98; 95% CI, 0.96–0.99; *p* = 0.032) as factors associated with traditional ESD technical success. In the multivariate analysis, rectal localization (OR, 3.23; 95% CI, 1.65–6.32; *p* = 0.001), F1 fibrosis grade (OR, 2.16; 95% CI, 1.10–4.26; *p* = 0.026), and previously biopsied lesions (OR, 0.33, 95% CI, 0.59–0.89; *p* = 0.006) were found to be independent predictors for avoiding conversion to H-ESD (Table 5). Univariate analysis used to identify predictors of cR and CR did not show any statistically significant correlation (Supplementary Table S3).

## 4. Discussion

The management of NLCLs remains one of the most challenging scenarios in therapeutic endoscopy. Nonetheless, endoscopic techniques offer a valuable alternative to surgery for a significant number of patients. Our study highlights the effectiveness of the ESD/H-ESD approach, demonstrating favorable outcomes with an RR of 3.6% over a median follow-up of 373 days (IQR 540), despite the complex nature of the treated lesions. Notably, 4/6 recurrences observed during SC1 occurred following H-ESD. While H-ESD may carry a higher recurrence risk, it is crucial to consider its role as a rescue therapy for particularly challenging lesions that often require piecemeal resection. All recurrences were successfully managed with endoscopic re-treatment, and after SC2, no patient required additional treatments. Moreover, no AEs occurred during these re-treatments, highlighting the safety of the approach in experienced hands.

In terms of safety, our study demonstrated an AE rate of 13.5%, which is consistent with the complexity of treating NLCLs. The most common complication was perforation, occurring in 8.4% of cases, while bleeding was observed in 3.4% of procedures. Only 1.7% of patients experienced grade IIIa AEs (according to the AGREE classification), which are the most severe. These findings demonstrate that the ESD/H-ESD approach maintains safety profiles consistent with the literature [29,30]. None of the patients required surgical intervention for AEs; surgery was only indicated when histological evidence of invasive cancer was found. All intraprocedural complications were promptly recognized and managed endoscopically, underscoring the importance of adequate expertise. 

The TS rate in our study was 71.9%; while considering only traditional ESD, it was 62.4%. These rates are lower compared to in some studies in the literature, ranging from 80 to 96%, but they must be contextualized within several important factors [7,21,29,30]. First, our study spans from 2009, encompassing the evolution of ESD techniques and devices, including the introduction of the pocket creation method, the traction-assisted ESD, and the saline immersion ESD [31,32,33,34]. Additionally, ESD has a steep learning curve, and the progressive accumulation of operator experience likely contributed to improved success rates over time.

Despite these challenges, our CR rate of 93.0% and the low RR observed during follow-up highlight the effectiveness of ESD/H-ESD in achieving durable, curative outcomes, even when en bloc resection is not achieved. 

**Figure 4:**
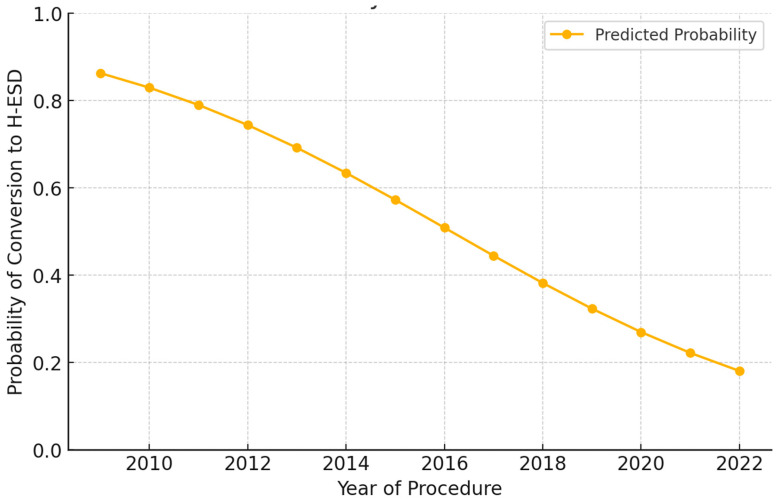
Trend of the predicted probability of conversion to hybrid endoscopic submucosal dissection (H-ESD) over time.

The temporal logistic regression analysis demonstrated a significant 22.7% annual reduction in the probability of conversion to H-ESD (OR = 0.7727; 95% CI: 0.6811–0.8765; *p* = 0.0001) (Figure 4). These findings indicate that as experience and technique refinement increased, the need for hybrid conversion progressively decreased. This trend reflects how the refinement of ESD methods, the integration of advanced dissection strategies, and the improvement of endoscopic tools have already enhanced ESD outcomes and represent a pathway toward even better results in the future. The multivariate analysis identified rectal localization, F1 fibrosis grade, and previously biopsied lesions as independent predictors of ESD TS without the need for hybrid conversion. The higher success rate in rectal lesions may be due to easier endoscopic access and stability. The association with F1 fibrosis and previously biopsied lesions is unsurprising, as milder, more localized fibrosis allows for easier submucosal lifting and dissection. These predictors can guide risk stratification and help identify cases where traditional ESD is most likely to succeed. 

Japanese multicenter studies have consistently demonstrated superior outcomes in ESD for NLCLs. A large retrospective study reported en bloc and CR rates of 95% and 90%, respectively, with a 6% perforation rate, over a 10-year period, and no local recurrences following cR [35]. Similarly, a prospective study showed a 96.3% en bloc and 83.3% CR rate, with a 7.4% perforation rate and no recurrence over a median follow-up of 60 months, further reinforcing the long-term efficacy of ESD in this setting [30]. This stark contrast highlights the persistent disparity in ESD outcomes between Eastern and Western centers, though AE rates remain comparable. Several factors may contribute to this difference. In Japan, higher case volumes and more structured, uniformly implemented training programs provide endoscopists with greater exposure and a more standardized learning pathway. The higher prevalence of gastric lesions allows trainees to first develop ESD skills on gastric cases before advancing to colorectal procedures. Additionally, the widespread adoption of standardized techniques ensures more consistent outcomes. In contrast, Western centers often face limitations in volume and training uniformity, leading to variability in technique adoption and a prolonged learning curve.

Our multicenter study, comprising 178 cases, represents one of the largest Western series addressing NLCLs through ESD. While Eastern centers report larger series, our cohort size surpasses most European studies and is comparable to recent large series (Table 6). This is particularly relevant given the absence of standardized treatment for NLCLs, where therapeutic strategies are largely dictated by operator expertise, center resources, and referral volumes. Various alternative techniques have been proposed.

Ablative techniques such as forced argon plasma coagulation (APC) and hot-biopsy avulsion have been proposed as potential treatment strategies. They are relatively straightforward to perform; however, they are associated with high RR (10–59%) and critically do not allow for histopathological assessment [30,36]. This limitation is significant, as the inability to assess for potential malignancy can lead to suboptimal treatment.

EMR represents a well-known alternative, yet the challenge of achieving adequate pre-resection lifting remains unresolved [37,38,39,40] and shows an RR up to 30% [37,41,42]. On the other hand, it has demonstrated a solid safety profile, with 6% incidence of bleeding, 2% incidence of intraprocedural perforation, and an even lower rate of delayed perforation [43,44].

Underwater EMR (U-EMR) offers advantages through reduced luminal distension and enhanced tissue capture due to increased mucosal buoyancy, even with fibrosis [45]. Its feasibility is suggested by limited retrospective evidence showing that U-EMR may achieve lower RRs compared to conventional EMR (11% vs. 66%), while maintaining a favorable safety profile [46]. When compared to ESD in a propensity-matched analysis, it demonstrated lower en bloc (73% vs. 100%) and CR rates (41% vs. 81%), with shorter hospital stays and a lower perforation rate, as expected [47].

Another approach, the cold avulsion with adjuvant snare-tip soft coagulation (CAST), offers a technically simple and valid option [48,49]. However, RRs of up to 5.0–18.8% have been described, and AEs are non-negligible, with an intraprocedural perforation rate of up to 5.6% and a need for clip application in almost 24% of procedures [48,49].

Furthermore, owing to their inability to achieve en bloc resection, the previous techniques necessitate close surveillance and potential repeated endoscopic treatments, thereby increasing the number of colonoscopies to which these patients are subjected. This represents a critical point, as the cumulative burden may further affect adherence to follow-up protocols.

On the other hand, endoscopic full-thickness resection (EFTR) allows for the en bloc resection of fibrotic lesions ranging 5–30 mm in size, depending on the tissue’s pliability [50]. However, its efficacy is limited by lesion size, with larger lesions (≥20 mm) demonstrating an R0 resection rate of only 58% [51,52]. Nevertheless, it is associated with an RR of 6.4–13.5%, and its applicability in the right colon is constrained by the device’s size and the consequent reduction in maneuverability [29,51,52,53,54]. As recently demonstrated by Yzet et al., ESD shows a lower RR compared to EFTR (2.1% vs. 11.9%, *p* = 0.09) even for lesions < 20 mm [29]. However, EFTR exhibits a better safety profile, with a lower rate of AEs (5.1% vs. 11.3%, *p* = 0.01). This highlights that ESD offers the best outcomes in terms of radicality and recurrence, albeit with a slightly higher risk of complications, while EFTR is indicated for smaller lesions, yields a non-negligible RR, and should be reserved for cases where ESD is not viable.

Our study has several strengths, including a large multicenter cohort with an extended follow-up period. The comprehensive analysis of TS predictors, coupled with the achieved low RR, provides valuable insights for patient selection and procedure planning in this challenging scenario

However, our study is not without limitations. The retrospective design and heterogeneous patient population may introduce potential biases. The lack of a control group with alternative techniques prevents definitive conclusions regarding the role of ESD in the management of NLCLs. Although all procedures were performed by experienced endoscopists, some degree of inter-operator variability cannot be excluded. This variability may also stem from differences in available equipment across centers, and distinct procedural habits, despite operators having comparable training backgrounds. Finally, since all procedures were conducted in high-volume tertiary centers by expert endoscopists, the generalizability of our findings to lower-volume centers or less experienced operators may be limited.

**Table 6:** Comparison of Western and Eastern studies on the treatment of NLCLs using endoscopic submucosal dissection (ESD).

Author	Year	Country	Study Design	Endoscopic Techniques	Patients (*n*)	Age (Mean, years)	Sex (Male, %)	Lesion Size Median (Range, mm)	Procedural Time (Median, min)	En Bloc Resection (%)	Adverse Events (%)
Hurlstone et al. [55]	2008	United Kingdom	Prospective monocentric	ESD	30	65	72	NA (5–45)	62	93%	16
Spychalski et al. [56]	2019	Poland	Retrospective monocentric	ESD	70	65	55.7	34.8 (23–46)	77	NA	12.8
Faller et al. [57]	2020	France	Retrospective multicentric	ESD	53	70	50.9	40 (20–65)	43	92.5	9.4
Yzet et al. [29] *	2023	France	Retrospective multicentric	ESD/eFTR	177	69	76	35 (5–140)	45	94.3	16.3
Tanaka et al. [35]	2021	Japan	Retrospective multicentric	ESD	102	69	55	20 (4–50)	84	95	8.8
Tanaka et al. [58]	2024	Japan	Prospective multicentric	ESD	54	70	63	31 (24.5–40)	65	96.3	9.5
Dell’Anna et al.	2025	Italy	Retrospective multicentric	ESD/H-ESD	178	68	56.2	30 (10–120)	80	71.9	13.4

* Data shown in the table refer only to the ESD-treated group of this study. ESD = endoscopic submucosal dissection; NLCL = non-lifting colorectal lesion; eFTR = endoscopic full-thickness resection; H-ESD = hybrid endoscopic submucosal dissection.

## 5. Conclusions

In conclusion, ESD and H-ESD as rescue therapy demonstrate feasibility, efficacy, and safety in treating NLCLs when performed by experienced endoscopists in tertiary referral centers. This approach yields a 3.6% RR, all amenable to successful endoscopic management in our experience. Factors associated with higher TS rates include previously biopsied lesions rather than recurrencies, rectal localization, and lower grades of fibrosis. These findings further support the potential of ESD as a treatment for these challenging lesions.

## Figures and Tables

**Table 1:** Baseline characteristics of the included patients with sub-analysis for fibrosis cause and technique.

	Overall (*n* = 178)	Previously Biopsied (*n* = 52)	Recurrencies (*n* = 126)	*p* Value	ESD (111)	H-ESD (67)	*p* Value
Age, mean (SD) (years) *	68.4 (11.3)	68.6 (11.8)	68.3 (11.1)	0.871	67.5 (10.6)	69.9 (12.1)	0.161
Sex, (*n*, %)				0.794			0.842
Female	78 (43.8)	22 (42.3)	56 (44.4)		48 (43.2)	30 (44.7)	
Male	100 (56.2)	30 (57.7)	70 (55.6)		63 (56.7)	37 (55.2)	
ASA (*n*, %) **				0.683			0.322
I-II	153 (87.4)	43 (82.7)	110 (87.3)		93 (83.8)	60 (89.6)	
III	25 (12.6)	9 (17.3)	16 (12.7)		18 (16.2)	7 (10.4)	
Localization (*n*, %) *				0.804			<0.001
Rectum	90 (50.6)	24 (46.2)	66 (52.4)		69 (62.1)	21 (31.3)	
Left colon	25 (14.0)	7 (13.5)	18 (14.3)		14 (12.6)	11 (16.4)	
Transverse colon	17 (9.6)	5 (9.6)	12 (9.5)		9 (8.1)	8 (11.9)	
Right colon	46 (25.8)	16 (30.8)	30 (23.8)		19 (17.1)	27 (40.3)	
Fibrosis degree (*n*, %) **			15 (11.9)	<0.01			<0.001
F1	34 (19.1)	19 (36.5)	111 (88.1)		31 (27.9)	3 (4.5)	
F2	144 (66.9)	33 (63.5)			80 (72.1)	64 (95.5)	
Paris morphology (*n*, %) **				0.696			0.925
Is/IIa + Is	92 (51.7)	25 (48.1)	67 (47.6)		58 (52.3)	34 (50.7)	
IIa	84 (47.2)	26 (50.0)	58 (46.0)		52 (46.8)	32 (47.8)	
IIb/IIc	2 (1.1)	1 (1.9)	1 (0.8)		1 (0.9)	1 (1.5)	
LST morphology (*n*, %) **				0.535			0.508
Granular	44 (31.4)	15 (37.5)	29 (29.0)		30 (27.0)	14 (20.9)	
Non granular	41 (29.2)	13 (32.5)	28 (28.0)		23 (20.7)	18 (26.9)	
Mixed	55 (39.2)	12 (30.0)	43 (43.0)		32 (28.8)	23 (34.3)	
Kudo classification (*n*, %)							
III/IV	151 (84.8)	35 (67.3)	116 (92.1)	<0.01	89 (80.2)	62 (92.5)	0.026
Vi	27 (15.2)	17 (32.7)	10 (7.9)		22 (19.8)	5 (7.5)	
Dimension long axis, mm (median, IQR) ***	30 (20)	35 (20)	30 (20)	0.006	30 (21)	30 (20)	0.161
Dimension short axis, mm (median, IQR) ***	20 (15)	25 (20)	20 (15)	0.035	25 (20)	20 (15)	0.037
Area, cm^2^ × 0.25π (median, IQR) ***	4.9 (7.1)	7.1 (9.1)	4.7 (6.3)	0.011	6.8 (10.1)	4.7 (7.1)	0.021
Procedure time, min (median, IQR) **	80 (60)	85 (69)	79.5 (65)	0.044	30 (60)	82 (60)	0.647
Knife type (*n*, %) ***							0.271
Hybrid type	125 (70.2)	29 (55.7)	96 (76.1)		79 (71.2)	46 (68.7)	
Dual type	39 (21.9)	22 (42.3)	17 (13.5)	<0.001	26 (23.4)	13 (19.4)	
Hook type	14 (7.9)	1 (1.9)	13 (10.3)		6 (5.4)	8 (11.9)	
ESD technique (*n*, %) ***				0.459			0.018
Standard	167 (93.8)	47 (90.4)	120 (95.2)		100 (90.1)	67(100)	
Tunnel/Pocket	11 (6.2)	5 (9.6)	6 (4.8)		11 (9.9)	0 (0)	
Traction (*n*, %)				0.137			
Yes	10 (5.6)	5 (9.6)	5 (4.0)		10 (90)	0 (0)	0.007
No	168 (94.4)	47 (90.4)	121 (96.0)		101 (10)	67 (100)	
Dissection speed, mm^2^/min (median, IQR) *	6.1 (8.1)	7.0 (10.8)	6.1 (7.3)	0.057	7.5 (8.8)	5.2 (5.2)	0.018

ESD = endoscopic mucosal dissection; H-ESD = hybrid endoscopic mucosal dissection. * Normally distributed variables were tested with Student’s *t*-test. ** Non-significant also at Bonferroni correction. *** Non-normally distributed variables were tested with Mann–Whitney U test.

**Table 2:** Lesion recurrence at surveillance colonoscopies with outcomes, stratified for fibrosis cause and ESD/H-ESD procedure.

	Early Recurrence			Late Recurrence		
	SC1			SC2		
	Recurrence	Treatment	Histology	Recurrence	Treatment	Histology
Fibrosis cause (*n*, %)						
Previously biopsied	1 (16.7)	EMR 0	LGD 0	1 (50)	EMR 1 (50)	LGD 1 (50)
		HBA 0	HGD 1 (16.7)		HBA 0	HGD 0
		ESD 0	Other 0		ESD 0	Other 0
		H-ESD 1 (16.7)			H-ESD	
		FTR 0			FTR 0	
Recurrent	5 (83.3)	EMR 2 (33.3)	LGD 5 (83.3)	1 (50)	EMR 0	LGD 1 (50)
		HBA 2 (33.3)	HGD 0		HBA 0	HGD 0
		ESD 1 (16.7)	Other 0		ESD 1 (50)	Other 0
		H-ESD 0			H-ESD 0 (50)	
		FTR 0			FTR 0	
Procedure (*n*, %)						
ESD	2 (33.3)	EMR 1 (16.7)	LGD 2 (33.3)	1 (50)	EMR 1 (50)	LGD 1 (50)
		HBA 1 (16.7)	HGD 2 (33.3)		HBA 0	HGD 0
		ESD 1 (16.7)	Other 0		ESD 0	Other 0
		H-ESD 1 (16.7)			H-ESD 0	
		FTR 0			FTR 0	
H-ESD	4 (66.7)	EMR 1 (16.7)	LGD 2 (33.3)	1 (50)	EMR 0	LGD 1 (50)
		HBA 1 (16.7)	HGD 0		HBA 0	HGD 0
		ESD 0	Other 0		ESD 1 (50)	Other 0
		H-ESD 0			H-ESD 0	
		FTR 0			FTR 0	
Overall (*n*, %)	6 (100)	EMR 2 (33.3)	LGD 5 (70)	2 (100)	EMR 1 (50)	LGD 2 (100)
		HBA 2 (33.3)	HGD 1 (30)		HBA 0	HGD 0
		ESD 1 (16.7)	Other 0		ESD 1 (50)	Other 0
		H-ESD 1 (16.7)			H-ESD 0	
		FTR 0			FTR 0	

SC = surveillance colonoscopy; EMR = endoscopic mucosal resection; HBA = hot-biopsy avulsion; ESD = endoscopic mucosal dissection; H-ESD = hybrid endoscopic mucosal dissection; FTR = full-thickness resection; LGD = low-grade dysplasia; HGD = high-grade dysplasia.

**Table 3:** Outcomes and safety, according to fibrosis cause.

	Overall (*n* = 178)	Previously Biopsied (*n* = 52)	Recurrencies (*n* = 126)	*p* Value
Technical success/en bloc resection (*n*, %)				0.004
Yes	128 (71.9)	43 (82.7)	85 (67.5)	
No	60 (28.1)	9 (17.3)	41 (32.5)	
ESD technical success (*n*,%)				0.04
Yes	111 (62.4)	41 (78.8)	70 (55.6)	
No	67 (37.6)	11 (21.2)	56 (44.4)	
CR * rate (*n*, %)				0.475
Yes	119 (93.0)	39 (90.7)	80 (94.1)	
No	9 (7.0)	4 (9.3)	5 (5.9)	
cR * rate (*n*, %)				0.475
Yes	119 (93.0)	39 (90.7)	80 (94.1)	
No	9 (7.0)	4 (9.3)	5 (5.9)	
Histology (*n*, %) **				0.277
LGD/HGD	153 (86.0)	41 (78.9)	112 (88.9)	
ADK (sm1)	11 (6.1)	5 (9.6)	6 (4.7)	
ADK (sm2)	10 (5.6)	5 (9.6)	5 (4.0)	
Other	4 (2.3)	1 (1.9)	3 (2.4)	
Early adverse events (*n*, %) **				0.197
Perforation	15 (8.4)	6 (11.5)	9 (7.1)	
Bleeding	5 (2.8)	0 (0)	5 (4.0)	
Both	1 (0.6)	1 (1.9)	0 (0)	
Late adverse events (*n*, %) **				
Perforation	0 (0)	0 (0)	0 (0)	
Bleeding	3 (0.017)	2 (0.04)	1 (0.008)	
Surgery (*n*, %)				0.134
Yes	11 (6.1)	5 (9.6)	6 (4.7)	
Histology	11 (6.1)	5 (9.6)	6 (4.7)	
Adverse event	0 (0)	0 (0)	0 (0)	
Follow-up time, days (median, IQR)	373 (540)	401 (358)	360 (648.2)	0.11

ESD = endoscopic mucosal dissection; cR = curative resection; CR = complete resection; LGD = low-grade dysplasia; HGD = high-grade dysplasia; ADK = adenocarcinoma. * The total and the statistics refer to all cases of excluded piecemeal resections, not comprisable in the category of neither CR nor cR. ** Non-significant at Bonferroni correction as well.

**Table 4:** Histological and safety outcomes of ESD versus H-ESD converted procedures.

	ESD (111)	H-ESD (67)	*p* Value
Technical success/en bloc resection *(n*, %)			
En Bloc	107 (96.4)	21 (31.3)	<0.001
Piecemeal	4 (3.6)	46 (68.7)	
Fibrosis cause (*n*, %)			
Previously biopsied	41 (36.9)	11 (16.4)	<0.004
Recurrent	70 (63.1)	56 (83.6)	
Histology (*n*, %) *			
LGD/HGD	90 (81.1)	63 (94.0)	0.249
ADK (sm1)	9 (8.1)	2 (3.0)	
ADK (sm2)	8 (7.2)	2 (3.0)	
Other	4 (3.6)	0 (0.0)	
cR ** rate (*n*, %)			
Yes	98 (91.9)	20 (95.2)	0.656
No	9 (8.1)	1 (4.7)	
CR ** rate (*n*, %)			
Yes	98 (91.9)	20 (95.2)	0.656
No	9 (8.1)	1 (4.7)	
Adverse events (*n*, %) *			
Perforation	10 (9.9)	5 (7.4)	0.532
Bleeding	4 (5.4)	1 (1.4)	
Both	1 (0.9)	0 (0)	
Delayed perforation	0 (0)	0 (0)	
Delayed bleeding	3 (2.7)	0 (0)	
Follow-up time, days (median, IQR)	376.0 (438.7)	366.0 (815.5)	0.55

ESD = endoscopic mucosal dissection; H-ESD = hybrid endoscopic mucosal dissection; LGD = low-grade dysplasia; HGD = high-grade dysplasia; ADK = adenocarcinoma; cR = curative resection; CR = complete resection. * Non-significant at Bonferroni correction as well. ** The total and the statistics refer to all cases of excluded piecemeal resections, not comprisable in the category of neither CR nor cR.

**Table 5:** Predictors of ESD technical success at univariate and multivariate analyses.

	Univariate		Multivariate	
	Unadjusted Odds Ratio (95% CI)	*p* Value	Odds Ratio (95% CI)	*p* Value
Localization (*n*, %) Rectum Left colon Transverse colon Right colon	0.10 (0.03–0.28)	0.001	0.10 (0.03–0.28)	<0.001
Fibrosis degree (*n*, %) F1 F2	0.12 (0.03–0.44)	0.0017	0.09 (0.02–0.40)	0.0003
Fibrosis cause (*n*, %) Previously biopsied Recurrent	0.33 (0.58–0.86)	0.004	0.33 (0.59–0.89)	0.006
Paris morphology (*n*, %) Is/IIa-Is IIa Other	1.21 (0.80–1.83)	0.351		
LST morphology (*n*, %) ** Granular Non granular Mixed	0.97 (0.78–1.21)	0.822		
Kudo classification (*n*, %) III/IV Vi	0.41 (0.17–1.00)	0.051		
Dimension long axis, mm (median, IQR) ***	0.98 (0.96–0.99)	0.032	1.01 (0.98–1.03)	0.397
Dimension short axis, mm (median, IQR) ***	0.98 (0.96–1.01)	0.125		
Area, cm^2^ × 0.25π (median, IQR) ***	0.98 (0.95–1.01)	0.199		

LST = laterally spreading tumor. ** Total refers to the 85 non-sessile (0-Is) lesions. *** Non-normally distributed variables were tested with the Mann–Whitney U test.

## Data Availability

The original contributions presented in the study are included in the article, further inquiries can be directed to the corresponding author.

## Supplementary Materials

The following supporting information can be downloaded at https://www.mdpi.com/article/10.3390/cancers17071242/s1: Table S1. STROBE statement—checklist; Table S2. AGREE classification grading definition; Table S3. Additional baseline characteristics of the included patients with sub-analysis for fibrosis cause; Table S4. Numbers of patients included in each center and endoscopic submucosal dissection (ESD) to hybrid ESD (H-ESD) conversion per center; Table S5. Technical unsuccess causes; Table S6. AGREE classification according to the type of endoscopic procedure; Table S7. Univariate analysis of complete resection (CR) and curative resection (cR) rates. Table S8. Endoscopic submucosal dissection (ESD) and hybrid ESD (H-ESD) conversion across study periods (2009–2022). Figure S1. Scatter plot illustrating the relationship between dissection speed (mm^2^/min) and the regression standardized predicted value.
