# Supplementary material for: Endoscopic Submucosal Dissection (ESD) for the Management of Fibrotic Non-Lifting Colorectal Lesions (NLCLs): Results from a Large Multicenter Retrospective Study
Source: Cancers (Basel). 2025 Apr 6;17(7):1242. doi: 10.3390/cancers17071242 (PMC11987842; doi:10.3390/cancers17071242)

Supplementary Table 1. STROBE Statement—Checklist.

	Item No	Recommendation
**Title and abstract**	1	(*a*) Indicate the study's design with a commonly used term in the title or the abstract
		(*b*) Provide in the abstract an informative and balanced summary of what was done and what was found
**Introduction**		
Background/rationale	2	Explain the scientific background and rationale for the investigation being reported
Objectives	3	State specific objectives, including any prespecified hypotheses
**Methods**		
Study design	4	Present key elements of study design early in the paper
Setting	5	Describe the setting, locations, and relevant dates, including periods of recruitment, exposure, follow-up, and data collection
Participants	6	(*a*) Give the eligibility criteria, and the sources and methods of selection of participants. Describe methods of follow-up
		(*b*) For matched studies, give matching criteria and number of exposed and unexposed
Variables	7	Clearly define all outcomes, exposures, predictors, potential confounders, and effect modifiers. Give diagnostic criteria, if applicable
Data sources/ measurement	8	For each variable of interest, give sources of data and details of methods of assessment (measurement). Describe comparability of assessment methods if there is more than one group
Bias	9	Describe any efforts to address potential sources of bias
Study size	10	Explain how the study size was arrived at
Quantitative variables	11	Explain how quantitative variables were handled in the analyses. If applicable, describe which groupings were chosen and why
Statistical methods	12	(*a*) Describe all statistical methods, including those used to control for confounding
		(*b*) Describe any methods used to examine subgroups and interactions
		(*c*) Explain how missing data were addressed
		(*d*) If applicable, explain how loss to follow-up was addressed
		(*e*) Describe any sensitivity analyses
**Results**		
Participants	13	(a) Report numbers of individuals at each stage of study—eg numbers potentially eligible, examined for eligibility, confirmed eligible, included in the study, completing follow-up, and analysed
		(b) Give reasons for non-participation at each stage
		(c) Consider use of a flow diagram
Descriptive data	14	(a) Give characteristics of study participants (eg demographic, clinical, social) and information on exposures and potential confounders
		(b) Indicate number of participants with missing data for each variable of interest
		(c) Summarise follow-up time (eg, average and total amount)
Outcome data	15	Report numbers of outcome events or summary measures over time
Main results	16	(*a*) Give unadjusted estimates and, if applicable, confounder-adjusted estimates and their precision (eg, 95% confidence interval). Make clear which confounders were adjusted for and why they were included
		(*b*) Report category boundaries when continuous variables were categorized
		(*c*) If relevant, consider translating estimates of relative risk into absolute risk for a meaningful time period
Other analyses	17	Report other analyses done—eg analyses of subgroups and interactions, and sensitivity analyses
**Discussion**		

Key results	18	Summarise key results with reference to study objectives
Limitations	19	Discuss limitations of the study, taking into account sources of potential bias or imprecision. Discuss both direction and magnitude of any potential bias
Interpretation	20	Give a cautious overall interpretation of results considering objectives, limitations, multiplicity of analyses, results from similar studies, and other relevant evidence
Generalisability	21	Discuss the generalisability (external validity) of the study results
**Other information**		
Funding	22	Give the source of funding and the role of the funders for the present study and, if applicable, for the original study on which the present article is based

Supplementary Table 2. AGREE Classification grading definition

No adverse event	• A telephone contact with the general practitioner, outpatient clinic, or endoscopy service without any intervention or<br>• Extended observation of the patient after the procedure, <3hours without any intervention
Grade I	Adverse events with any deviation of the standard post-procedural course, without the need for pharmacologic treatment or endoscopic, radiologic or surgical interventions.<br>• Presentation at the emergency ward, without any intervention or<br>• Hospital admission (<24 hours), without any intervention or<br>• Allowed therapeutic regimens are drugs as antiemetics, antipyretics, analgesics, and electrolytes or<br>• Allowed diagnostic tests: radiology and laboratory tests
Grade II	• Adverse events requiring pharmacologic treatment with drugs other than those allowed for grade I adverse events (ie, antibiotics, antithrombotics, etc) or<br>• Blood or blood product transfusions or<br>• Hospital admission for more than 24 hours
Grade III	Adverse events requiring endoscopic, radiologic, or surgical intervention
Grade IIIa	Endoscopic or radiologic intervention
Grade IIIb	Surgical intervention
Grade IV	Adverse events requiring intensive care unit/critical care unit admission
Grade Iva	Single-organ dysfunction (including dialysis)
Grade IVb	Multiorgan dysfunction
Grade V	Death of the patient

Supplementary table 3. Additional baseline characteristics of the included patients with sub-analysis for fibrosis cause.

	Overall (n=178)	***Previously biopsied (n=52)***	***Recurrencies (n=126)***	***P value***
**Anticoagulants (n,%)**				
Yes	14 (7.1)	6 (11.5)	8 (6.3)	0.242
No	164 (82.8)	46 (88.5)	118 (93.7)	
**Anti-platelet agent (n,%)**				
Yes	30 (15.2)	12 (23.1)	18 (14.3)	0.154
No	148 (74.7)	40 (76.9)	108 (85.7)	
**Diabetes, (n,%)**				
Yes	13 (6.6)	8 (15.4)	5 (4.0)	0.008
No	165 (83.3)	44 (84.6)	121 (96.0)	
**Cardiovascular disease, (n,%)***				
Yes	151 (76.3)	10 (19.2)	17 (13.5)	0.332
No	27 (13.2)	42 (80.8)	109 (86.5)	

Supplementary table 4. Numbers of patients Included in each center and endoscopic submucosal dissection (ESD) to Hybrid-ESD (H-ESD) conversion per center

(n,%)	*Center 1*	*Center 2*	*Overall*	*p-value*
ESD	52 (46.8)	15 (22.4)	67 (37.6)	<0.001
H-ESD	59 (53.2)	52 (77.6)	111 (62.4)	

Supplementary table 5. Technical failure causes.

**Technical failure cause (n,%)**	
Severe Fibrosis	42 (62.7)
Difficult endoscopic access	19 (28.3)
Hemodynamic instability	2 (3.0)
Perforation	4 (6.0)

Supplementary Table 6. AGREE Classification according to the type of endoscopic procedure.

Grading	*ESD (111)*	*H-ESD (67)*	*Overall (178)*	*p-value*
No adverse event (n, %)	94 (84.6)	60 (89.5)	154 (86.5)	0.348
Grade I	5 (4.5)	1 (1.4)	6 (3.3)	0.234
Grade II	11 (9.9)	5 (7.4)	16 (8.9)	0.780
Grade III	2 (1.8)	1 (1.5)	3 (1.7)	
Grade IIIa	2	1	3	1
Grade IIIb	0	0	0	
Grade IV	0	0	0	
Grade IVa				-
Grade IVb				
Grade V	0	0	0	-

Supplementary Table 7. Univariate analysis of Complete Resection (CR) and and curative Resection (cR) rate

	CR*		Univariate	cR*		Univariate
	Yes (n=119)	No (n=9)	P value	Yes (n=119)	No (n=9)	P value
**Localization (n,%)****						
Rectum	65 (54.6)	6 (66.7)	0.759	65 (54.6)	6 (66.7)	0.759
Left colon	14 (11.8)	1 (11.1)		14 (11.8)	1 (11.1)	
Right/Transverse colon	40 (33.6)	2 (22.2)		40 (33.6)	2 (22.2)	
**Fibrosis degree (n,%)****						
F1	31 (26.1)	2 (22.2)	0.953	31 (26.1)	2 (22.2)	0.953
F2	88 (73.9)	5 (77.8)		88 (73.9)	5 (77.8)	
**Paris morphology (n, %)****						
Is/IIa-Is	63 (52.9)	2 (22.2)	0.170	63 (52.9)	2 (22.2)	0.170
IIa	54 (45.4)	7 (77.8)		54 (45.4)	7 (77.8)	
Other	2 (1.7)	0 (0)		2 (1.7)	0 (0)	
**LST morphology^{+} (n, %)****						
Granular	33 (27.7)	1 (11.1)	0.277	33 (27.7)	1 (11.1)	0.277
Non granular	25 (21,0)	4 (44.4)		25 (21,0)	4 (44.4)	
Mixed	30 (25.2))	3 (33.3)		30 (25.2))	3 (33.3)	
**Dimension long axis, mm (median, IQR)*****	30 (20)	30 (43)	0.198	30 (20)	30 (43)	0.198
**Dimension short axis, mm (median, IQR)*****	21 (20)	20 (19)	0.784	21 (20)	20 (19)	0.784
**Area, cm^{2}x0.25π (median, IQR)*****	5.2 (8.6)	7.1 (10.8)	0.849	5.2 (8.6)	7.1 (10.8)	0.849
**Knife type (n,%)****						
Hybrid type	81 (68.1)	5 (55.6)	0.727	81 (68.1)	5 (55.6)	0.727
Dual type	30 (25.2)	3 (33.3)		30 (25.2)	3 (33.3)	
Hook type	8 (6.7)	1 (11.1)		8 (6.7)	1 (11.1)	
**ESD technique^{++} (n,%)****			0.065			0.065

Standard<br>*Tunnel*<br>*Pocket*	110 (92.4)<br>5 (4.2)<br>4 (3.3)	7 (77.7)<br>2 (22.2)<br>0 (0)		110 (92.4)<br>5 (4.2)<br>4 (3.3)	7 (77.7)<br>2 (22.2)<br>0 (0)	
**Traction (n,%)**<br>*Yes*<br>*No*	110 (92.4)<br>9 (7.5)	9 (100.0)<br>0 (0)	0.392	110 (92.4)<br>9 (7.5)	9 (100.0)<br>0 (0)	0.392
**Fibrosis cause (n,%)**<br>Previously biopsied<br>Recurrent	39 (32.7)<br>80 (67.2)	4 (44.4)<br>5 (55.5)	0.475	39 (32.7)<br>80 (67.2)	4 (44.4)<br>5 (55.5)	0.475

* The total and the statistics refer to all cases excluded piecemeal resections, not comprisable into the category of neither CR and cR
**Not significant also at Bonferroni correction
***Non normally distributed variables were tested with Mann-Whitney U test
+ Definable only for Lateral spreading tumors
++ Definable only for ESD successfully treated lesions

Supplementary table 8. Endoscopic submucosal dissection (ESD) and Hybrid-ESD (H-ESD) conversion across study periods (2009–2022).

Period (n, %)	***ESD (n=111)***	***H-ESD (n=67)***	***Overall (n=178)***	***p-value***
2009-2015	13 (41.9)	18 (58.1)	31 (17.5)	0.0117
2016-2022	98 (66.6)	49 (33.3)	147 (82.5)	

Supplementary figure 1. Scatter plot illustrating the relationship between dissection speed (mm²/min) and the regression standardized predicted value.

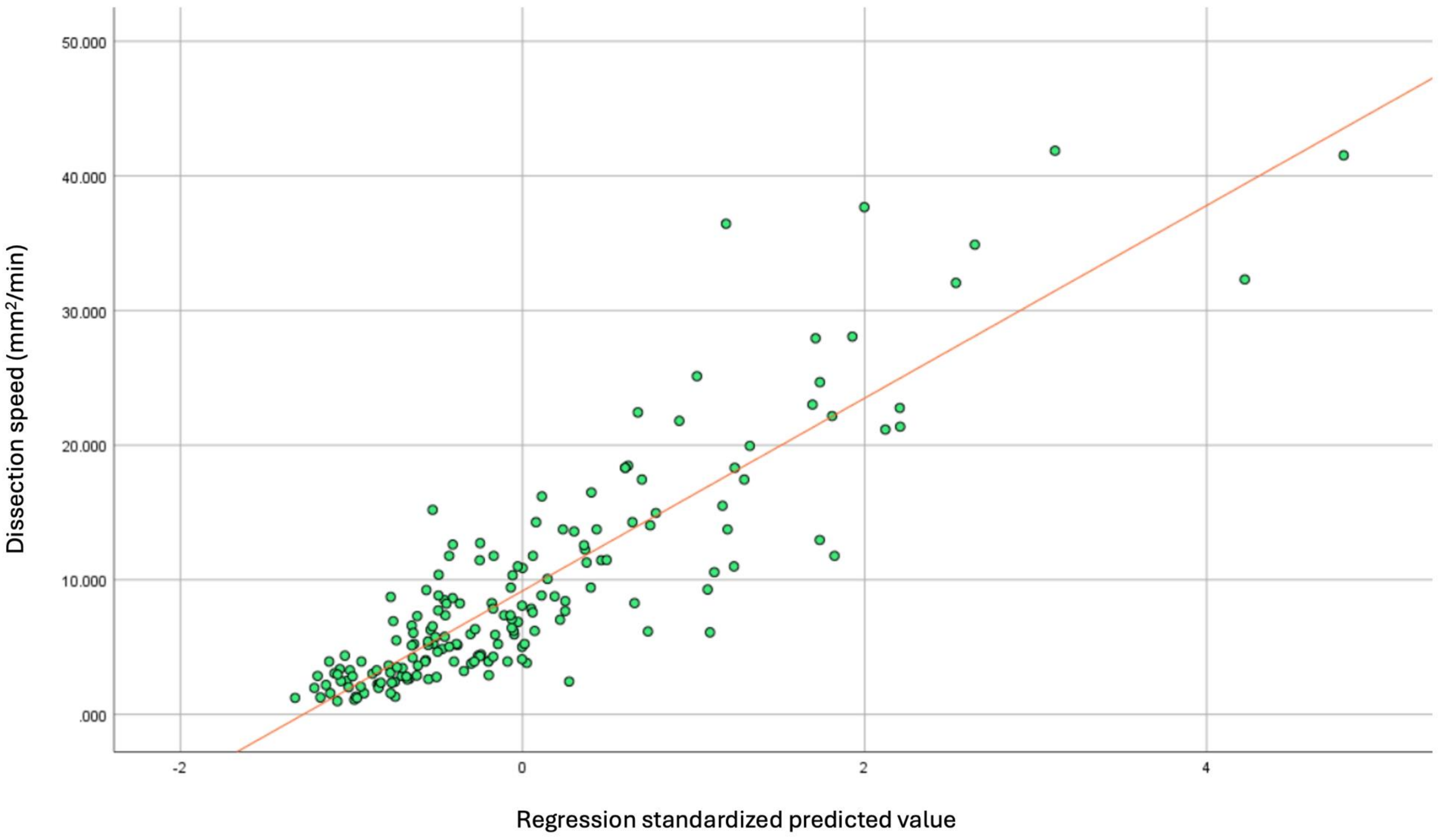

Supplement: Supplementary file 1 [file cancers-17-01242-s001.zip › cancers-3459907-supplementary.pdf]
